# Bacterial Cytolysin during Meningitis Disrupts the Regulation of Glutamate in the Brain, Leading to Synaptic Damage

**DOI:** 10.1371/journal.ppat.1003380

**Published:** 2013-06-13

**Authors:** Carolin Wippel, Jana Maurer, Christina Förtsch, Sabrina Hupp, Alexandra Bohl, Jiangtao Ma, Timothy J. Mitchell, Stephanie Bunkowski, Wolfgang Brück, Roland Nau, Asparouh I. Iliev

**Affiliations:** 1 DFG Membrane/Cytoskeleton Interaction Group, Institute of Pharmacology and Toxicology & Rudolf Virchow Center for Experimental Medicine, University of Würzburg, Würzburg, Germany; 2 Division of Infection and Immunity, Glasgow Biomedical Research Centre, University of Glasgow, Glasgow, United Kingdom; 3 Chair of Microbial Infection and Immunity, School of Immunity and Infection, College of Medical and Dental Sciences, University of Birmingham, Birmingham, United Kingdom; 4 Department of Neuropathology, Georg-August-University of Göttingen, Göttingen, Germany; 5 Department of Geriatrics, Evangelisches Krankenhaus Göttingen-Weende, Göttingen, Germany; University of California, San Francisco, United States of America

## Abstract

*Streptococcus pneumoniae* (pneumococcal) meningitis is a common bacterial infection of the brain. The cholesterol-dependent cytolysin pneumolysin represents a key factor, determining the neuropathogenic potential of the pneumococci. Here, we demonstrate selective synaptic loss within the superficial layers of the frontal neocortex of post-mortem brain samples from individuals with pneumococcal meningitis. A similar effect was observed in mice with pneumococcal meningitis only when the bacteria expressed the pore-forming cholesterol-dependent cytolysin pneumolysin. Exposure of acute mouse brain slices to only pore-competent pneumolysin at disease-relevant, non-lytic concentrations caused permanent dendritic swelling, dendritic spine elimination and synaptic loss. The NMDA glutamate receptor antagonists MK801 and D-AP5 reduced this pathology. Pneumolysin increased glutamate levels within the mouse brain slices. In mouse astrocytes, pneumolysin initiated the release of glutamate in a calcium-dependent manner. We propose that pneumolysin plays a significant synapto- and dendritotoxic role in pneumococcal meningitis by initiating glutamate release from astrocytes, leading to subsequent glutamate-dependent synaptic damage. We outline for the first time the occurrence of synaptic pathology in pneumococcal meningitis and demonstrate that a bacterial cytolysin can dysregulate the control of glutamate in the brain, inducing excitotoxic damage.

## Author Summary

Bacterial meningitis is one of the most devastating brain diseases. Among the bacteria that cause meningitis, *Streptococcus pneumoniae* is the most common. Meningitis predominantly affects children, especially in the Third World, and most of them do not survive. Those that do survive often suffer permanent brain damage and hearing problems. The exact morphological substrates of brain damage in *Streptococcus pneumoniae* meningitis remain largely unknown. In our experiments, we found that the brain cortex of patients with meningitis demonstrated a loss of synapses (the contact points among neurons, responsible for the processes of learning and memory), and we identified the major pneumococcal neurotoxin pneumolysin as a sufficient cause of this loss. The effect was not direct but was mediated by the brain neurotransmitter glutamate, which was released upon toxin binding by one of the non-neuronal cell types of the brain – the astrocytes. Pneumolysin initiated calcium influx in astrocytes and subsequent glutamate release. Glutamate damaged the synapses via NMDA-receptors – a mechanism similar to the damage occurring in brain ischemia. Thus, we show that synaptic loss is present in pneumococcal meningitis, and we identify the toxic bacterial protein pneumolysin as the major factor in this process. These findings alter our understanding of bacterial meningitis and establish new therapeutic strategies for this fatal disease.

## Introduction

*Streptococcus pneumoniae* (pneumococcal) meningitis is the most common form of bacterial meningitis [1]. The patient survival rate is 75%; however, half of the patients suffer from long-term disabilities [2]. This disease is associated with significant but not massive neuronal death, predominantly in the hippocampus [3].

Pneumolysin (PLY) is a critical pneumococcal pathogenic factor that belongs to the cholesterol-dependent cytolysin (CDC) group. This 54-kD protein contains four domains and targets plasmalemmal cholesterol through its fourth domain [4]. Upon binding, PLY oligomerizes into pre-pore rings of 30–50 monomers. Subsequently, domain 3 of each monomer refolds and penetrates the membrane, transforming the pre-pore to a pore [5]. Multiple cellular toxic effects (e.g., small GTPase activation and microtubule stabilization), although pore-dependent, occur at sub-lytic and non-lytic concentrations, which is indicative of a more complex interaction between the toxin and cells [6]–[8]. To obtain tools to study the cellular effects of pore formation by PLY, the delta6 mutant form of PLY has been created [9], which lacks the amino acids alanine 146 and arginine 147. This mutation makes the refolding of domain 3, and thus pore formation, impossible [9]. PLY is critical for the clinical course of experimental pneumococcal meningitis [10], [11], and PLY-deficient *S. pneumoniae* bacteria initiate a substantially milder disease course. PLY is persistent in the cerebrospinal fluid (CSF), which correlates with a poorer prognosis in human patients [12]. However, the mechanism of this PLY dependence remains largely unclear. There is some experimental evidence from a rabbit model, however, that argues against the role of PLY in meningitis [13], raising the question of the specificity of different animal models.

In bacterial meningitis, pathogenic bacteria multiply in the CSF and are abundant adjacent to the white matter (along the ventricles) and the neocortex. The neocortex is a highly specialized and complex brain structure comprised of up to six layers. Its major component is pyramidal neurons (∼80% of all cortical neurons are pyramidal), whose somata are positioned predominantly in layers III, V and VI, send projections throughout the cortex and receive projections from other neurons from other brain areas. Particularly complex and dense synaptic connections are established in the superficial layers I–II. Positioned within the neocortex among the pyramidal neurons, multiple interneurons (typically spiny stellate and basket cells) establish shorter connections with neighboring neurons. Generally, the pyramidal neurons are described as glutamatergic, with some exceptions (for review see [14]).

Synapses are complex structures, consisting of a pre- and post-synapse and the surrounding cells (such as astrocytes) [15]. Astrocytes wrap around the synaptic cleft and parts of the pre- and/or post-synapse, thereby allowing them to rapidly remove released neurotransmitters [16]. The morphological structures that host the active post-synapses along the dendrites are the dendritic spines [17]. The dendritic spines are dynamic; not all of them host synapses, but all synapses of pyramidal neurons are positioned on spines [18]. Long-term potentiation or depression are associated with spine growth or shrinkage [19]. The permanent loss and alteration of dendritic spines correlate with decreased synaptic numbers and cognitive impairment in multiple neurological disorders [20], [21].

Our aim was to study changes in neocortical synapses as a result of *Streptococcus pneumoniae* meningitis and the role of the major pneumococcal neurotoxin PLY in producing these effects.

## Results

To address the question of synaptic damage resulting from meningitis, we used four experimental approaches: i) human frontal neocortical brain samples from autopsy cases; ii) a pneumococcal meningitis mouse model (3–5 month-old C57Bl/6 mice); iii) an acute mouse brain slice model (postnatal day 12–14 C57Bl/6 mice); and iv) a primary mouse astrocyte culture system.

### Reduction of synapses in human forebrain postmortem samples from meningitis patients

**Figure 1:**
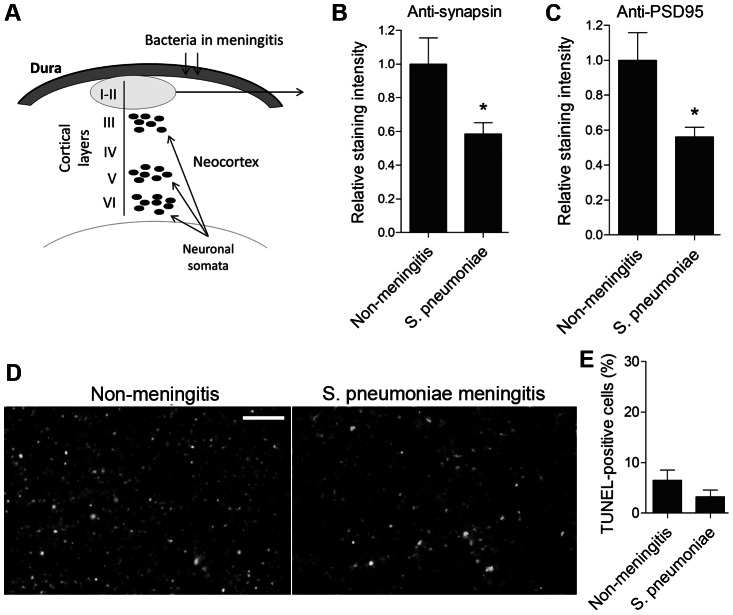
Reduced synaptic density in human postmortem pneumococcal meningitis neocortical brain tissue samples. *A.* Schematic representation of the analyzed neocortical regions. *B*, *C.* Decreased synapsin I (*B*) and the PSD95 (*C*) staining densities in layers I–II of the frontal neocortex of human post-mortem samples from *S. pneumoniae* meningitis cases (S. pneumoniae) *vs.* post mortem samples of cases who experienced rapid non-neurological death (Non-meningitis). *D.* Representative tissue samples (layer II) with anti-synapsin I immunohistochemistry. Scale bar: 10 µm. *E.* There was no difference in the number of TUNEL-positive nuclei in neocortical layers I–II between non-meningitis and meningitis samples. All values represent the mean ± SEM, and samples from 5 to 6 cases per group were analyzed.

Human frontal neocortical brain samples obtained from autopsy cases following death from *S. pneumoniae* meningitis and from control cases following a rapid death from non-neurological causes (the exact descriptions of the patient groups are presented in Table 1 and Table 2) were stained with anti-synapsin I (a pre-synaptic marker protein [22]) and anti-PSD95 (post-synaptic density 95; a post-synaptic protein, specific for glutamatergic synapses [23]) antibodies. In the superficial neocortical layers I–II, pre- and post-synaptic immunostaining levels were significantly reduced in the meningitis-infected samples (Fig. 1A–D). The total cell density and the number of dying cells (measured by TUNEL staining for apoptotic cells) were identical between the groups within the area of analysis (Fig. 1E). The presence of TUNEL-positive cells in both groups may be due to the post-mortem delay until sample collection. In all slices, the analyzed cortical areas excluded necrotic regions, which were present in some patient samples. Thus, *S. pneumoniae* meningitis induced a reduction in synaptic density that was independent of cell death.

**Table 1:** Clinical histories of the individual patients from the non-meningitis histology group.

Case Number	Age, sex	Underlying disease(s)	Immediate cause of death
1	59, m	COPD	Pulmonary failure/shock
2	60, m	None	Chest and abdominal trauma
3	82, f	Diarrhea, hypertension, aortic valve replacement, cardiac failure	Aspiration
4	42, m	Myocardial infarction, CAD	Ventricular fibrillation
5	58, f	Coronary heart disease, myocardial infarction	Ventricular fibrillation
6	66, f	Renal insufficiency, suspected pulmonary embolism	Acute cardiac failure

No focal necroses were present in the frontal cortical and hippocampal sections of control cases. In the hippocampal formation of case 58, f hypoxic injury was noted on HE stains.

ARDS: adult respiratory distress syndrome, CAD: coronary artery disease, COPD: chronic obstructive pulmonary disease.

**Table 2:** Clinical histories of the individual patients from the *Streptococcus pneumoniae* meningitis histology group.

Case Number	Age, sex	Immediate cause of death	Interval between onset of symptoms and death (days)	Other neuropathological abnormalities
1	56, f	Shock	12	Severe brain edema, herniation, necrosis of cortical and cerebellar neurons
2	81, f	Respiratory arrest	8	Moderate brain edema
3	61, f	Pulmonary embolia	6	Severe brain edema, herniation, diffuse neuronal necrosis neurons
4	63, f	Shock	91	Cortical microabscesses and focal necroses, watershed infarctions
5	57, m	Shock	6	Severe brain edema, hypoxic brain injury
6	54, m	Brain death	1	Brain edema, herniation

Note: the synaptic/cell death analysis in our tissue samples involved non-necrotic tissue areas.

### PLY-dependent reduction of synapses in the neocortex of pneumococcal meningitis mice

**Figure 2:**
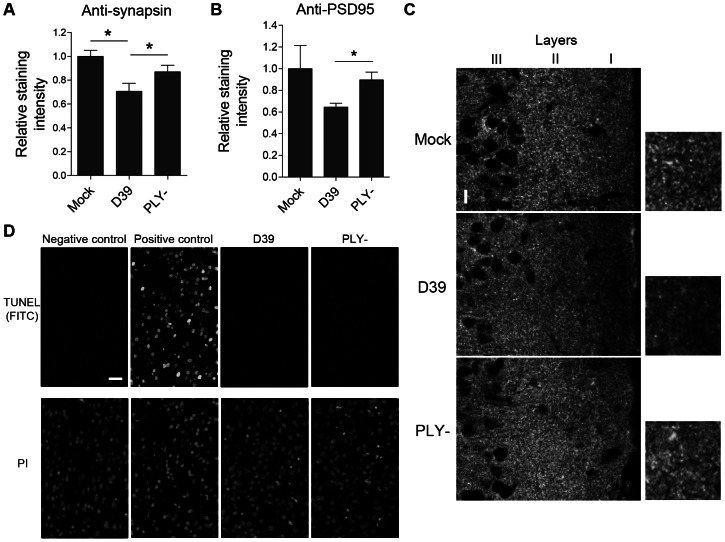
Reduced synaptic density in mouse pneumococcal meningitis neocortical brain tissue samples. *A.* Reduced synapsin staining in layers I–II of the neocortex in animals with meningitis by PLY-producing bacteria *vs.* all other groups 36 h after injection. * p<0.05. (D39) indicates the group of mice injected intracerebrally with the pneumolysin (PLY)-producing D39 *S. pneumoniae* strain; (PLY-) mice indicates those infected with the PLY-deficient D39 strain. *B.* Reduced staining was observed for PSD95 in layers I–II of the frontal neocortex of mice injected with the PLY-producing strain *vs.* the PLY-deficient D39 strain animals after 36 h. All values are presented as the mean ± SEM. There were 5 animals in the mock group and 10–13 in the meningitis group. *C.* Representative tissue sample images with anti-synapsin I immunohistochemistry of layers I–III with magnification of equivalent areas of interest in layer I. Scale bar: 15 µm. *D.* Representative images of the TUNEL-FITC staining of equivalent areas in layers I/II of the neocortex of mice infected with D39 and PLY-deficient pneumococcal strain, where no TUNEL-positive cells are present. All nuclei were counterstained with propidium iodide (PI). TUNEL-negative control (enzyme missing) and TUNEL-positive control (pretreatment with DNAseI) are presented for staining validation. Scale bar: 20 µm.

Next, we focused on the role of individual key pathogenic components of *S. pneumoniae* as synaptotoxic factors. We infected the right frontal brain lobes of C57BL/6 animals with the PLY-producing *S. pneumoniae* strain D39 and with PLY-deficient mutants of the D39 strain. Thirty-six hours after infection, we sacrificed the animals and stained paraffin brain slices in a manner similar to the human samples described above. There was a significant decrease in pre-synaptic (synapsin I) and post-synaptic (PSD95) staining in layers I–II of the frontal brain neocortex (Fig. 2A–C). There were virtually no TUNEL-positive cells in any of the slices from either group; we used DNAseI-treated slices as a positive control, showing 100% positive nuclear staining (Fig. 2D). These experiments confirmed that PLY deficiency strongly diminished synaptic loss in mouse pneumococcal meningitis without an increase in cell death.

### Selective synapto- and dendritotoxic effects of PLY in acute mouse brain slices

**Figure 3:**
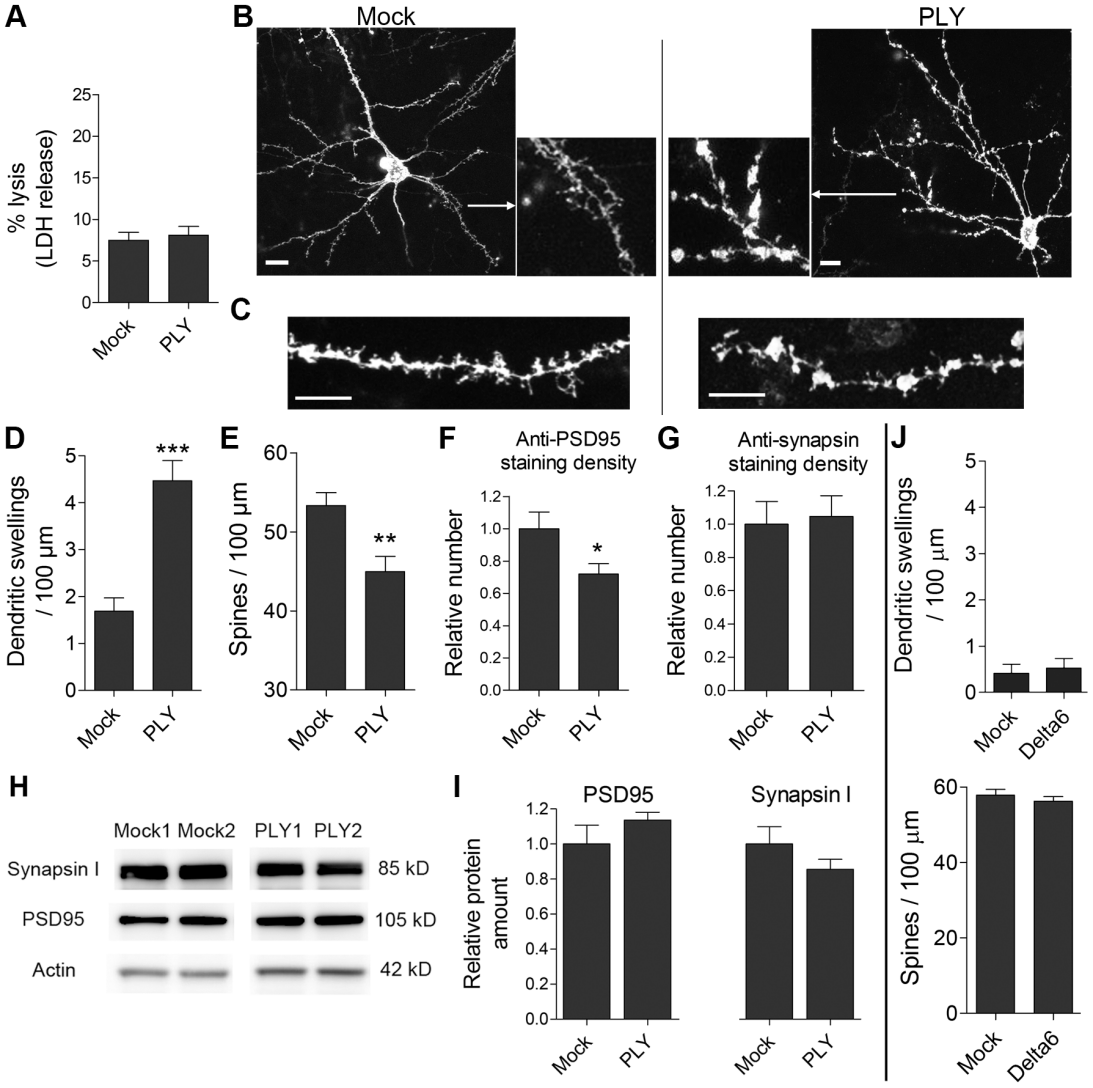
Dendritic and synaptic changes caused by pneumolysin in acute brain slices. *A.* Equivalent cell lysis (LDH release) between slices that were mock treated or treated with 0.2 µg/ml PLY for 8 h. *B.* A DiI-stained pyramidal neuron in the neocortex of an acute mouse slice demonstrated a normal spine and dendrite morphology (mock) in contrast to a PLY-treated slice (0.2 µg/ml for 5 h), which showed a reduction in spine number and multiple dendritic enlargements (swellings). Scale bars: 10 µm. *C.* Magnified dendritic fragments, demonstrating the dendrite configuration and the morphology of the dendritic spines. Scale bars: 10 µm. *D.* Increased number of dendritic swellings after exposure to 0.2 µg/ml PLY for 5 h. *** p<0.001. *E.* Decreased number of dendritic spines following 5 h of exposure to 0.2 µg/ml PLY. ** p<0.01. *F.* Reduced number of PSD95-positive fluorescent puncta in the neocortices of slices treated with 0.2 µg/ml PLY for 5 h. * p<0.05. *G.* Unchanged number of synapsin I-positive fluorescent puncta in the neocortices of slices treated with 0.2 µg/ml PLY for 5 h. *H.* Western blot analysis of the protein levels of synapsin I, PSD95 and actin in acute mouse brain slices treated with 0.2 µg/ml PLY for 5 h or in mock-treated slices. *I.* Unchanged protein expression levels of synapsin I and PSD95 in acute mouse brain slices (normalized to the corresponding levels of actin). *J.* The delta6 non-pore forming mutant of PLY did not produce varicosity increase and dendritic spine loss. All values are presented as the mean ± SEM; n = 6 slices from at least 3 independent experiments.

To clarify whether PLY alone can cause the observed synaptic changes, we studied the role of PLY at non-lytic, disease-relevant concentrations in acute mouse brain slice cultures as described previously [24]. We chose 12–14 day-old infant mice as the tissue source because they can be sliced with minimum cytotoxicity (<5%) and be maintained for up to 24 h in an oxygenized environment without losing viability, while already demonstrating signs of maturation (such as myelination) that resemble the normal tissue environment in human meningitis. At 0.2 µg/ml, PLY did not cause increased cell lysis compared with mock-treated slices (Fig. 3A). The cortex neurons were stained with the fluorescent stain DiI (see the Materials and Methods section) to visualize the neuronal bodies, dendritic trees and dendritic spines. Morphologically, ∼80% of the stained neurons were pyramidal, and ∼20% were non-pyramidal, corresponding to the normal proportion of these cell types in the neocortex. DiI stains only the dendritic tree of intact cells with non-interrupted neurites, as it dissolves into the soma and diffuses laterally throughout membrane lipids. Following a minimal PLY exposure of 5 h, the dendrites of acute slices from infant mice showed the formation of swellings (or varicosities; Fig. 3B–D) and a loss of dendritic spines (we defined dendritic spines according to the criteria in [25]; Fig. 3C, E). Similarly, the number of PSD95-positive structures in layers I–II of the neocortex of the acute mouse brain slices was diminished following 5 h of PLY exposure (Fig. 3F); however, the density of the synapsin I-positive structures was not affected (Fig. 3G). We observed no differences in the PSD95 and synapsin I expression levels in neocortical protein extracts between the mock and the PLY groups, which indicated that the loss of PSD95 synaptic staining was not due to decreased protein expression, but to elimination of post-synapses and protein redistribution (Fig. 3H, I). In contrast to the pore-competent toxin, the non-pore-forming delta6 mutant failed to cause any changes in spine number or dendritic swellings (Fig. 3J), confirming a critical role for the pore-forming properties, even at sub-lytic concentrations.

**Figure 4:**
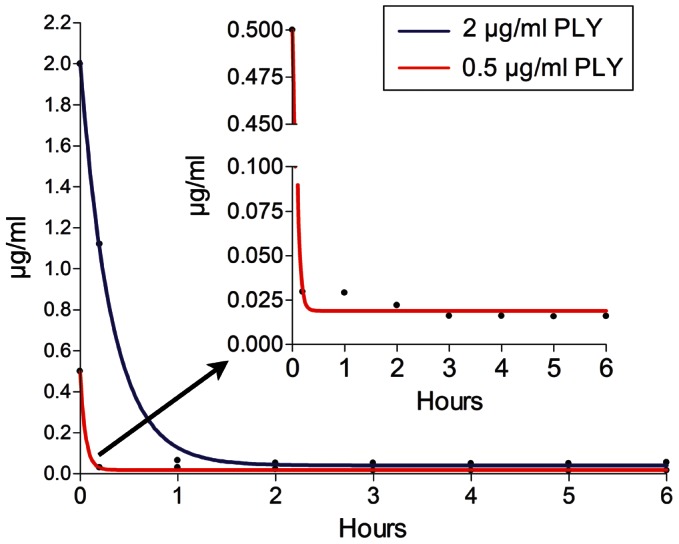
Kinetics of toxin tissue binding. Measurement of the fluorescence intensity of GFP-tagged PLY (PLY) in the medium following incubation of brain slices (6 slices per well) challenged with either 0.5 or 2 µg/ml PLY-GFP. The initial toxin concentration in the medium was high but rapidly (within minutes) decreased due to tissue binding. In the enlarged diagram (upper right), a rescaled y-axis fragment of the 0.5 µg/ml PLY experiment is presented.

To determine whether the PLY effects were reversible, it was important to clarify whether PLY bound tissue rapidly within a short period of time or slowly and continuously within the whole exposure time. Exposure of brain slices to PLY-GFP resulted in nearly complete toxin binding and depletion of the toxin from the medium within minutes (Fig. 4). This confirms that the dendritic swellings, spine collapse and synaptic loss detected after a 5-h PLY exposure were not short-term phenomena due to continuous binding of new toxin fractions, but long-term changes following an initial short toxic insult.

### The observed PLY effects were NMDA dependent

**Figure 5:**
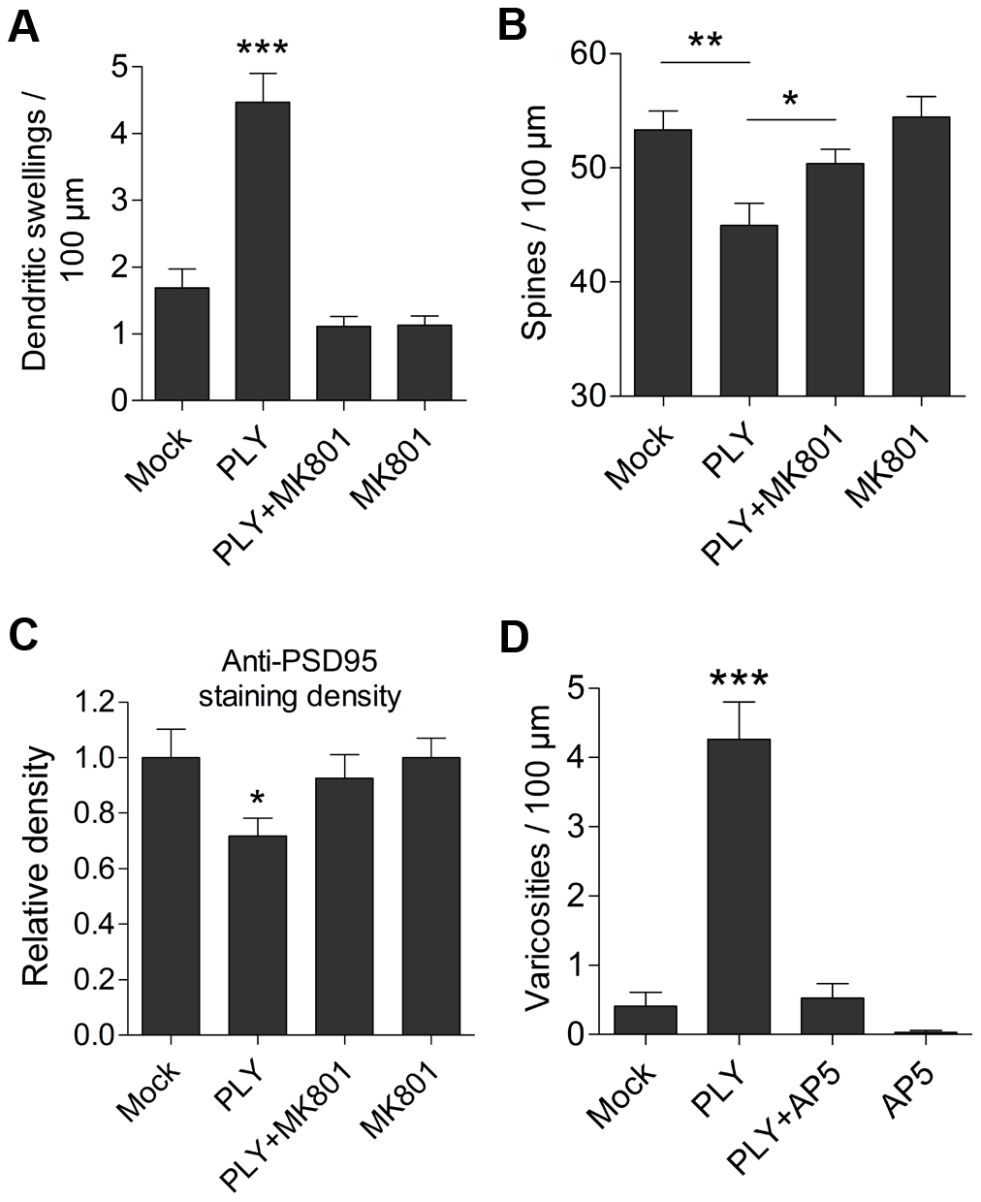
NMDA dependence of the dendritic changes caused by pneumolysin. *A.* Inhibition of the formation of dendritic swellings caused by treatment with 0.2 µg/ml PLY for 5 h by the application of 10 µM of the non-competitive NMDA-receptor inhibitor MK801. *** p<0.001 vs. all. *B.* Preserved dendritic spine number following treatment with 10 µM MK801 together with 0.2 µg/ml PLY for 5 h. * p<0.05, ** p<0.01. *C.* Reversal of the PSD95 density loss by PLY when incubated with 10 µM MK801. *D.* Complete inhibition of dendritic swelling formation caused by treatment with 0.2 µg/ml PLY for 5 h using a 50 µM of the competitive NMDA-receptor antagonist D-AP5. *** p<0.001. All values represent the mean ± SEM; n = 5–6 slices from at least 3 independent experiments.

The observed dendritic swellings closely resembled the effects of excitotoxicity on neurons in both primary cultures and brain slices, as observed by other laboratories [26]. Thus, we exposed acute mouse brain slices to 10 µM non-competitive NMDA inhibitor MK801 prior to the PLY challenge, and we observed a complete block of dendritic swelling formation (Fig. 5A) and a recovery of the number of dendritic spines in the neocortex (Fig. 5B). Similarly, PSD95 staining recovered to level of the control group (Fig. 5C). This effect was specific for NMDA receptors, as 50 µM competitive NMDA inhibitor D-AP5 also abolished the development of the PLY-induced dendritic and synaptic phenotype (Fig. 5D; the prevention of dendritic swelling formation is demonstrated as an example).

### PLY initiated increases in glutamate in brain tissue and around astrocytes in a calcium-dependent manner

**Figure 6:**
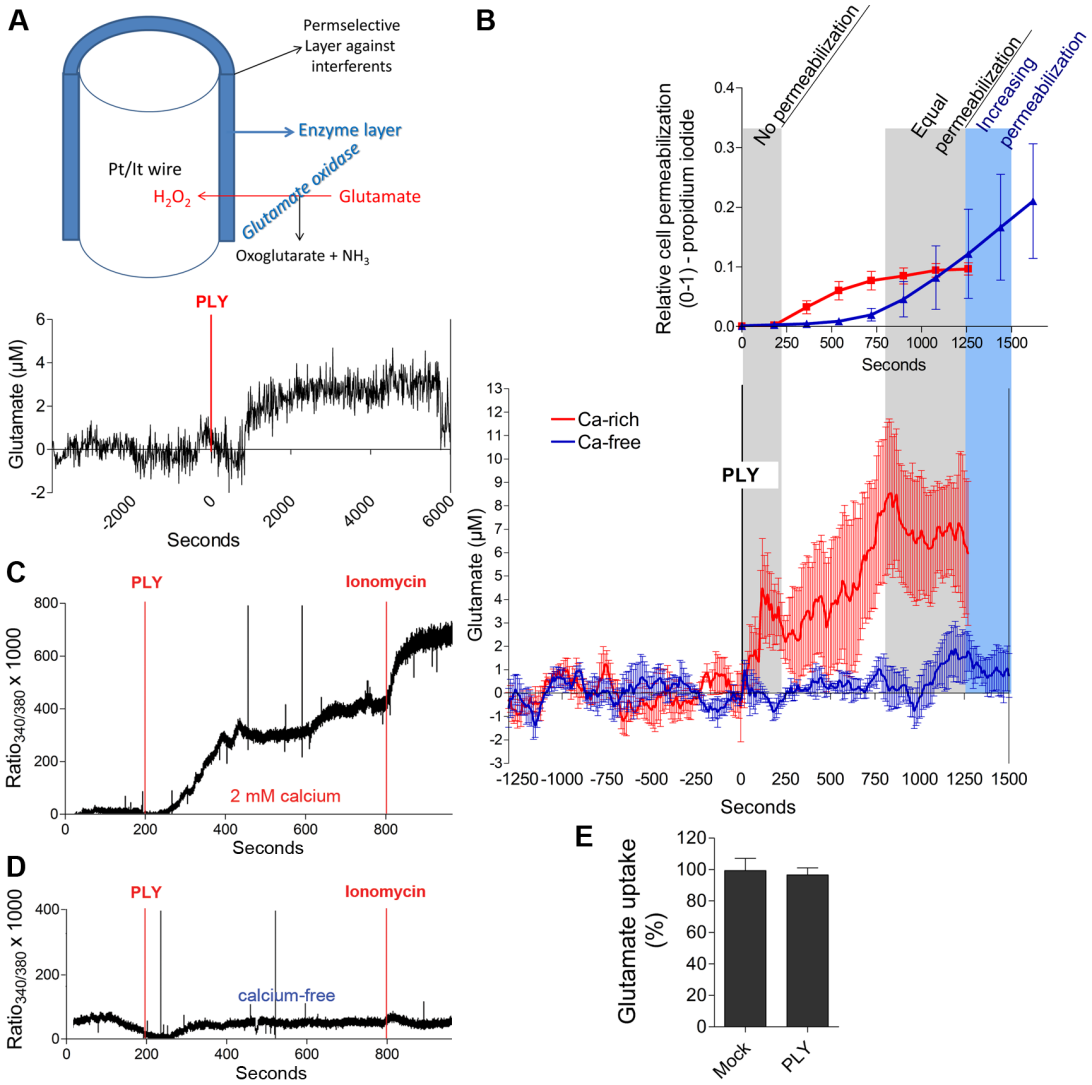
Increased glutamate release and calcium changes caused by pneumolysin. *A.* Representative sample of three experiments demonstrating increased neocortical glutamate content (via electrochemical detection in an acute slice; a diagram of the electrode is presented) following 0.2 µg/ml PLY exposure. *B.* Elevation of glutamate release on the surface of a monolayer of mouse astrocytes by a treatment with 0.1 µg/ml PLY in buffer containing 2 mM extracellular calcium (Ca-rich) *vs.* unchanged glutamate levels in calcium-free buffer (Ca-free). A permeabilization diagram (propidium iodide-positive cells) is presented above the glutamate release diagram. The values are presented as the means ± SEM; n = 3–5 experiments. *C.* Increase in cytosolic calcium (Fura-2-loaded mouse astrocytes) following treatment with 0.1 µg/ml PLY and a 10 µM ionomycin control at 800 s in 2 mM calcium-containing buffer (representative experiment). The experiments were repeated 5 times with identical results. *D.* Unchanged cytosolic calcium concentration following an identical incubation as in *C.*, but under calcium-free extracellular buffer conditions. *E.* Preserved glutamate uptake in brain slices following 0.2 µg/ml PLY challenge for 4 h; n = 3 experiments.

The excessive activation of NMDA receptors by the endogenous neocortical synapse mediator glutamate causes excitotoxicity [27]. We analyzed the occurrence of increased glutamate release using glutamate electrochemical sensors (Fig. 6A) in acute mouse brain slices following PLY exposure. The glutamate concentrations in the cortex increased to ∼3 µM above background levels within minutes of a PLY challenge (Fig. 6A). The glutamate source could be either the astrocytes or the synaptic structures. Considering the remodeling effects of PLY on astrocytes [8] and the fact that astrocytes are expected to be the primary structures in contact with the toxin before it reaches the synapses, we tested the possibility that sub-lytic PLY concentrations (concentrations inducing less than 10% cell lysis in the cultures and no lysis in slices) could induce glutamate release from cultured astrocytes. Primary mouse astrocytes were exposed to 0.1 µg/ml PLY, and glutamate levels were measured with an electrochemical sensor on the monolayer surface. Following an initial glutamate peak (before the first signs of permeabilization; Fig. 6B), glutamate levels increased further together with the increase in permeabilization (Fig. 6B). Glutamate release was completely blocked under calcium-free buffer conditions, as the observed cell lysis was identical or even greater compared with the cell lysis in 2 mM calcium conditions (Fig. 6B), which we have described previously [24]. The calcium-free experiments confirmed that permeabilization alone was not sufficient to increase the glutamate levels around the astrocytes and that calcium was required for this effect.

To verify that the cytosolic calcium increase by PLY was completely eliminated under calcium-free buffer conditions, we performed a Fura-2 analysis in astrocytes. Within 2–4 min of PLY exposure, the astrocytes demonstrated an intracellular calcium increase in medium containing 2 mM calcium (Fig. 6C; consistent with the increased glutamate release) that was fully eliminated under calcium-free buffer conditions (Fig. 6D). The uptake of glutamate from acute slices, which could also be responsible for the elevation in tissue glutamate, remained unaffected independent of PLY treatment (Fig. 6E).

## Discussion

In this study, we described for the first time the occurrence of synaptic loss in the neocortex in human pneumococcal meningitis autopsy cases and in an animal model of bacterial meningitis, and we identified PLY as a critical factor in this process. We further analyzed the cellular and molecular mechanisms of PLY-based dendritic and synaptic damage, confirming the importance of its pore-forming capacity, local glutamate release and the activation of NMDA receptors. PLY was capable of inducing glutamate release from astrocytes in a calcium-dependent manner, which we speculate to be a major source of elevated brain glutamate in pneumococcal meningitis and meningitis model systems.

The observed long-term cognitive decline in survivors of pneumococcal meningitis implies the occurrence of synaptic damage and loss as the reasons for cognitive abnormalities, although it has never been proven. Synaptic loss is present in multiple CNS diseases such as Alzheimer's, Huntington's, and Creutzfeldt-Jacob [20], [28], [29]. Synaptic loss correlates better with the level of cognitive decline than cell death and thus is suggested to be a major factor for the cognitive deterioration in these diseases [30]. Our findings demonstrate that synaptic loss is also present in infectious brain diseases such as pneumococcal meningitis. Furthermore, our data suggest a very compelling mechanism of cognitive decline in meningitis, although other pathogenic factors may also play a role.

Experiments on neonatal rats with pneumococcal meningitis have demonstrated the existence of a greater number of apoptotic cells in the hippocampus and cortex that increases in a PLY-dependent manner [10]. In contrast to the infant rat meningitis model, mouse meningitis models (similar to ours) are characterized by substantially lower levels of cortical cell death (if any) [31], [32], allowing for the precise analysis of dendritic and synaptic changes during the pathogenesis of pneumococcal meningitis. Thus, the observed selective synaptotoxic effect of PLY-producing D39 bacteria could be clearly separated from the loss of neuronal bodies, which did not occur in our mouse experiments.

Bacterial meningitis is a disease with a complex pathogenesis. Multiple additional factors (such as H_2_O_2_, CpG-DNA and others) may act synergistically with PLY in the process of synaptic damage. Thus, it was important to verify our findings regarding synaptic loss in mice with meningitis in another, PLY-based experimental setup. Therefore, we chose the acute mouse brain slice paradigm. This system allowed us to confirm that PLY alone was sufficient to cause synaptic loss and dendritic pathology.

The increased glutamate concentrations observed in the CSF of human bacterial meningitis cases correlates with disease severity [33], [34]. Spranger et al. assumed that the major source of glutamate in these CSF samples was the infiltrating monocyte population, which releases glutamate in the acute phase of the infection [34]. While the inflammatory background should be relatively similar in different types of bacterial meningitis (also assuming a similarity in the amounts of immune cell-derived glutamate), the local glutamate increase in the cortex following PLY exposure, which we observed, could act synergistically with the immune cell-derived glutamate. Brain tissue has very high glutamate uptake capacity [35]. The increase in the extracellular glutamate concentration within the cortices of our isolated slice system could be due either to a decreased uptake or an increased release. The experiments using glutamate-sensitive microsensors demonstrated that glutamate uptake by astrocytes remained unaffected following PLY exposure, while the release was increased. A similar pathogenic mechanism with increased glutamate release is involved in ischemic brain damage [36] and other neurological conditions [37]. The neurotoxic effect of glutamate, known as excitotoxicity, is widely studied in ischemic brain damage, although the patterns of excitotoxicity may differ depending on multiple additional factors such as the duration, persistence and glutamate amounts. The exact pathogenic mechanisms connecting the increased glutamate levels and the over-activation of NMDA receptors, calcium influx and subsequent dendritic alterations have been discussed extensively in the literature (for review see [27]) and fall beyond the scope of our work. In some cases where mitochondrial function is preserved and glutamate exposure is short, these swellings and dendritic spine losses are reversible within tens of minutes and contribute to better tissue adaptation and remodeling [38]. With an increased duration and strength of glutamate exposure, these changes become permanent and irreversible. The loss of glutamatergic synapse-specific PSD95 by PLY in our acute mouse brain slice system confirmed the permanent synaptic specificity of the PLY toxic insult. The rapid sequestration of PLY from the medium within minutes after slice exposure further confirms that the initial insult is short, and the effects 5 h later are rather long-term effects. Earlier studies that combined PSD95 staining and electrophysiology have confirmed that the loss of PSD95 completely correlates with the electrophysiological evidence for synapse elimination [39]. The reduction of synapsin I density in the brain meningitis samples (both mouse and human) but not in the PLY-treated acute mouse brain slices was most likely due to the difference in the analysis time points: 5 h for the acute slices and 36 h and more after the disease onset for the meningitis brain samples. Experimental evidence from other labs confirms that the reduction of PSD95 staining precedes the elimination of pre-synaptic markers and structures in excitotoxicity [39].

Both astrocytes and the glutamatergic presynaptic endings function as glutamate stores. The capacity of astrocytes to release glutamate from vesicular structures has previously been studied in detail [40]. This release occurs predominantly in a calcium-dependent manner, suggesting a possible mechanism utilized by PLY to increase glutamate release in our system [41], [42]. PLY is known to produce an increase in intracellular calcium concentrations [43]. In our experiments, we used lower, sub-lytic concentrations of PLY compared with those applied previously (we define “sub-lytic” as PLY concentrations producing less than 10% lysis in cell cultures and no lysis in the slice culture system) and again observed a calcium increase in all tested cells. Under calcium-free buffer conditions, the intracellular calcium levels remained unchanged following PLY challenge. This result confirmed that extracellular calcium is the principal source of the cytosolic calcium increase following PLY challenge under normal conditions [43]. The possibility of a secondary role for intracellular calcium stores could not be excluded, but it was not the initiating factor. In agreement with other studies of calcium dependence of glutamate release, the prevention of the intracellular calcium increase during the incubation of the astrocytes with PLY in a calcium-free buffer fully inhibited the elevation of extracellular glutamate. Thus, calcium influx by PLY was indeed the key event initiating glutamate release. Our experiments with astrocytes indicated their role as a primary glutamate source following PLY challenge. Nevertheless, synapses may be another source of PLY-induced glutamate release. However, considering the structural organization of the synaptic units, their ensheathing by astrocytes and the large surface area of astrocytes in the cortex, it appears reasonable to expect that astrocytes are the primary cellular component that encounter and are affected by PLY in the neocortex. In the astrocyte/glutamate experiments, we demonstrated the feasibility of a calcium-dependent glutamate release mechanism upon PLY challenge. We did not attempt to dissect the individual components of this mechanism, which can be even more complicated. It should be considered that autocrine mechanisms such as the triggering of ATP release and subsequent P2-receptor activation may also play a role in enhancing glutamate release [13].

An analysis of the calcium dependence of our findings in the slice cultures under calcium-free conditions was not an option due to the high calcium sensitivity of brain neurons in acute slices, which leads to the paradoxical formation of dendritic swellings under calcium-free conditions (not shown) and has also been observed by other researchers [44].

PLY has the capacity to modulate astrocyte shape by inducing actin remodeling [8], [45]. In these earlier works, PLY produced astrocyte shrinking and reorganization, increasing the penetration of toxic factors into the tissue, but not reducing the number of vital glial cells. There, despite the use of sub-lytic concentrations of PLY, the protein still requires an intact pore-forming capacity to remodel actin [45]. Similarly, we observed a pore-forming capacity dependence of PLY effects in both the varicosity number increase and spine loss phenotype experiments here, as the delta6 non-pore forming PLY protein was not able to alter the dendrites. While the actin remodeling effects by PLY on astrocytes do not require calcium [45], the release of glutamate, which is responsible for the synaptic and dendritic changes in our system, does. It is possible that calcium influx through the pores is responsible for the increase. In a broader perspective, both pathogenic mechanisms initiated by PLY, calcium-dependent and calcium-independent, may contribute to the complex brain tissue response to PLY.

An important question in our experimental acute brain slice system is the penetration capacity of PLY into the tissue. Earlier works of Braun et al. [46] confirm that PLY can penetrate the hippocampus. Our earlier experiments have also confirmed the ability of PLY to produce astrocyte shape changes deep into the cortical tissue, consistent with the areas of analysis here, as well as the ability of whole bacteria (substantially larger than the PLY molecule) and fluorescent dextran to penetrate deep into the cortex, a process that is further enhanced by PLY [47]. Histopathological brain samples from patients with pneumococcal meningitis also demonstrate the presence of intracortical microabscesses, which further increase the depth of PLY delivery into the cortical layers [48]. The breakdown of the blood-brain barrier, occurring early in bacterial meningitis, surely further promotes the delivery of toxic molecules into the brain parenchyma [49].

PLY is known to activate TLR4 and induce neuroimmunological responses [50], [51]. Detailed analyses by our group have demonstrated that at the applied non-lytic concentrations, PLY does not induce increased neuroinflammatory response within the initial 6–8 h of exposure, when most of the neuropathological effects of PLY occur [8]. Nevertheless, we cannot exclude that the neuroinflammatory alterations are operational at later time points.

Our studies demonstrate a pathogenic cascade linking the presence of PLY with glutamate release and the subsequent, NMDA-dependent dendritic and synaptic pathology associated with pneumococcal meningitis. During the course of bacterial meningitis, however, there is a plethora of complementary factors at work including oxidative stress, brain ischemia, edema, immune system-derived glutamate release, bacteria and bacterial products and other toxic factors. Thus, the role of PLY as a critical pro-inflammatory factor clearly contributes in a synergistic manner to all of the other factors (in parallel to the glutamate cascade we present here) to produce brain damage; however, we do not exclude the role of other factors, especially in human patients. In a broader context, the novel insight obtained herein regarding bacterial meningitis as a synaptic disease should be considered in future studies addressing the pathophysiology of meningitis.

## Materials and Methods

### Ethical statement

The animals used for tissue and cell culture preparation were handled in accordance with the regulations of the German Law for the Protection of the Animals, and the procedures were approved by the Ethics Committee of the Government of Lower Saxony. The human autopsy samples were obtained and used in accordance with the current German legal regulations including consent from the families of the deceased. Their use was approved by the Ethics Committee of the University Hospital Göttingen, Germany.

### PLY preparation

Wild-type PLY was expressed in *Escherichia coli* BL-21 cells (Stratagene, Cambridge, UK) and purified via metal affinity chromatography as described previously [52]. The purified PLY was tested for the presence of contaminating Gram-negative LPS using the colorimetric LAL assay (KQCL-BioWhittaker, Lonza, Basel, Switzerland). All purified proteins showed <0.6 endotoxin units/µg of protein. The initial stock of purified wild-type toxin exhibited an activity of approximately 5×10^4^ hemolytic units/mg. One hemolytic unit was defined as the minimum amount of toxin needed to lyse 90% of 1.5×10^8^ human erythrocytes per ml within 1 hr at 37°C. A second toxin stock with a slightly lower lytic activity was applied at corrected concentrations that were equivalent to the activity of the first stock and are expressed as the microgram equivalent throughout the text. The non-toxic delta6 version of the plasmid was constructed using site-directed mutagenesis (Quikchange SDMKit, Stratagene). The non-pore forming delta6 mutant (deletion of the amino acids alanine 146 and arginine 147; diagram shown in Fig. 1B) was produced with the following deletion primers: 5′-GGTCAATAATGTCCCAATGCAGTATGAAAAAATAACGGCTC-3′ and 5′-GAGCCGTTATTTTTTCATACTGCATTGGGACATTATTGACC-3.

The properties of this mutant were examined in detail previously [9], [45]; it binds comparably with the wild-type PLY but cannot perform the transition to a pore (Fig. 1B).

### Cell and slice cultures and culture treatments

Primary mouse astrocytes were prepared from the cortices of newborn C57BL/6 mice (postnatal day 3–5) as mixed cultures with microglia in Dulbecco's modified Eagle's medium (high glutamate; Gibco, Invitrogen GmbH, Karlsruhe, Germany). The growth medium was supplemented with 10% fetal calf serum (FCS; PAN Biotech GmbH, Aidenbach, Germany) and 1% penicillin/streptomycin (Gibco). Fourteen days after seeding in 75-cm^2^ cell culture flasks (Sarstedt AG & Co KG, Nuembrecht, Germany), the cells were reseeded.

The cells were treated with pneumolysin (PLY) in serum-free medium depending on the experiment. In the glutamate measurement and Fura-2 experiments, the cells were exposed to the toxin in a buffer containing 135 mM NaCl, 2.5 mM MgCl_2_, 4 mM KCl, and 5 mM Hepes (all from Carl Roth GmbH+Co. KG, Karlsruhe, Germany), pH 7.3, with or without 2 mM CaCl_2_.

Acute brain slices were prepared from infant (postnatal day 12–14) C57Bl/6 mice via decapitation and vibratome sectioning (Vibroslice NVSL, World Precision Instruments, Berlin, Germany) in artificial CSF continuously oxygenized with carbogen gas (95% O_2_, 5% CO_2_) at 4°C. The slices were allowed to adapt in carbogenated BME (GibcoBRL) with 1% penicillin/streptavidin and 1% glucose at 37°C for 1 h before being challenged with PLY in the 5% CO_2_-buffered medium (pH = 7.3). In these acute slices, cell lysis never exceeded 5% within 12 h.

### Mouse meningitis model

The model was performed exactly as described previously [53]. Briefly, 3 to 5 month-old anesthetized (ketamine [100 mg/kg of body weight] and xylazine [10 mg/kg]) C57BL/6 mice were infected in the right frontal lobe with 25 µl of 0.9% NaCl containing 10^4^ CFU of the different bacterial strains. The mice were followed up 36 h after infection, at which time, the mice were sacrificed by decapitation, and blood was collected. The cerebellum and the ventral half of the spleen were homogenized in 0.9% saline (1/10 [wt/wt]) to determine bacterial titers. The bacterial titer in cerebellar homogenates, although slightly higher in the D39 strain meningitis samples, did not differ significantly between the groups (not shown). The remaining brain tissue was fixed in 4% paraformaldehyde. For a more detailed assessment of neuronal damage, tissue samples from mice infected with D39 wild-type and pneumolysin-deficient *S. pneumoniae* (n = 10 each) were perfused with phosphate-buffered saline and 4% formalin and then embedded in paraffin. One-micrometer-thick coronary sections were further stained immunohistochemically and with TUNEL.

### Human pathological brain material

Neocortical brain samples from human autopsy cases were embedded in paraffin and analyzed. The individuals included in the control group (6 cases) all died from acute, non-neurological causes. The average age of the control group was 61 (range, 42–82), and this group included 3 males and 3 females. The meningitis victims (6 cases) suffered from microbiologically confirmed *S. pneumoniae*, which was the immediate cause of death. The average age of the meningitis group was 62 (range, 53–81), and this group included 4 females and 2 males. Detailed patient histories are presented in Table 1 and Table 2.

### Tissue staining

Acute mouse brain slices were fixed in 2% paraformaldehyde (Carl Roth) in PBS (pH 7.3) for 30 min, permeabilized with 0.1% Triton X-100 (Carl Roth), and processed either for immunohistochemistry or for neuron-specific DiI staining [54]. Briefly, crystals of the lipophilic DiI stain (Invitrogen) were positioned on layers IV–VI of the cortex and allowed to diffuse along the membranes of neurons with an intact neuritic tree, staining the apical dendrites and branches of pyramidal neurons. As only projecting branches with clear morphological characteristics were followed, shorter branched cells positioned close to the crystal (presumably not neurons) were avoided.

For the immunohistochemical experiments, microtome slices from mouse and human brain samples embedded in paraffin were deparaffinized and rehydrated, and the antigen was revealed using target retrieval solution (Dako Deutschland GmbH, Hamburg, Germany) at 95°C. The primary antibodies used were rabbit anti-MAP2 (1∶200; Santa Cruz Biotechnology, Inc., Heidelberg, Germany), mouse anti-synapsin I (1∶500; Synaptic Systems GmbH, Göttingen, Germany) and rabbit anti-PSD95 (Abcam, Cambridge, UK), and the secondary antibodies were goat anti-rabbit and goat anti-mouse antibodies tagged with FITC, Cy3 or Cy5 (1∶200; Dianova GmbH, Hamburg, Deutschland). The sample nuclei were stained with DAPI (4,6-diamidino-2-phenylindole dihydrochloride; Invitrogen; 1∶1000 in PBS). All samples were preserved with the ProLong antifade reagent (Invitrogen).

DNA fragmentation staining in the nuclei was detected using a DeadEnd Fluorometric TUNEL kit according to the manufacturer's instructions (Promega GmbH, Mannheim, Germany), and nuclear counter-staining was performed with propidium iodide (Invitrogen). Briefly, deparaffinized slices were rehydrated, treated with proteinase K (Promega) for 15 min and incubated with the reaction enzyme mix. Control slices were treated with DNase I (Qiagen) prior to labeling with terminal deoxynucleotidyl transferase to yield 100%-positive staining (positive control), and slices with no terminal deoxynucleotidyl transferase added served as a negative control and showed no staining.

All samples were analyzed either on an Olympus Cell M imaging fluorescent system (Olympus Europe, Hamburg, Germany) using a 20× dry objective or a 60× oil immersion objective or on a Leica SP5 laser-scanning microscope (Leica Microsystems Heidelberg GmbH, Mannheim, Germany) using a 63× oil immersion objective and with a zoom of up to 8x. Image processing and analyses were performed using ImageJ software (version 1.43 for Windows, National Institutes of Health, Bethesda, Maryland, U.S.A.).

### Calcium imaging

We utilized staining with Fura-2 AM (Invitrogen) preloaded for 30 min in primary astrocytes at a concentration of 5 µM in imaging buffer, followed by washing for 30 min before proceeding with imaging on an IonOptix microscopy system with a 63× oil immersion objective and analysis with IonWizzard software (all from IonOptix Limited, Dublin, Ireland).

### Glutamate measurement

Glutamate measurements were performed in slices and in cultures using glutamate oxidase-coated semipermeable microelectrodes (Sarissa Biomedical Limited, Coventry, United Kingdom), which detect glutamate based on an enzymatic transition and changes in the electric properties of the electrodes. The values obtained from a closely positioned identical reference electrode (without enzyme) were subtracted from those of the glutamate sensor to obtain the glutamate-specific signal; the electrodes were standardized at the end of the experiment against a known glutamate concentration. The slices, perfused with carbogen-enriched BME medium, or astrocytic cultures, incubated in imaging buffer, were analyzed in a tissue recording system equipped with micromanipulators (Kerr Scientific Instruments, Christchurch, New Zealand), and the signal was acquired with a potentiostat (Pinnacle Technology, Lawrence, Kansas, U.S.A.) and analyzed with Pal 8100 software (Pinnacle). The cultures were maintained at a temperature of 37°C. The measurements in the glutamate uptake experiments were performed in an identical manner with the addition of glutamate to a final concentration of 200 µM and subsequent measurements of the glutamate levels, which were normalized to background levels within 3 min.

### Protein biochemistry analysis

In Western blot experiments, samples containing equal amounts of proteins were electrophoresed on SDS-PAGE gradient gels (4–12% acrylamide, Anamed, Germany). The separated proteins were transferred via semi-dry blotting to PVDF Imobilion-P transfer membranes (Millipore, Germany). The membranes were blocked using 3–5% non-fat milk (Carl Roth) and were incubated with an anti-PSD95 rabbit antibody (Abcam Inc., Cambridge, MA USA; 1∶400), anti-synapsin I mouse antibody (Synaptic System GmbH, Göttingen, Germany; 1∶500) and anti-actin rabbit antibody (Cytoskeleton). Following incubation with a horseradish peroxidase-conjugated rabbit anti-mouse antibody (Jackson Immuno Research, USA), the signals were detected using ECL Plus Western Blotting Reagent (GE Healthcare, Munich, Germany) and a Fluorchem Q machine (Alpha Innotech GmbH, Kasendorf, Germany). Acute brain slices were mechanically homogenized by repetitive freeze-thaw cycles in liquid nitrogen followed by mechanical mixing, all in protease inhibitor mix (Roche Applied Science, Mannheim, Germany).

### Statistical analyses

Statistical analyses were performed using GraphPad Prism 4.02 for Windows (GraphPad Software Inc., La Jolla, CA, U.S.A.). The statistical tests included Mann-Whitney U-tests (comparing 2 groups differing in one parameter) and one-way ANOVA with a Bonferroni post-test (comparing 3 or more groups differing in one parameter).
